# IL-7 Up-Regulates TNF-α-Dependent Osteoclastogenesis in Patients Affected by Solid Tumor

**DOI:** 10.1371/journal.pone.0000124

**Published:** 2006-12-27

**Authors:** Ilaria Roato, Giacomina Brunetti, Eva Gorassini, Maria Grano, Silvia Colucci, Lisa Bonello, Lucio Buffoni, Roberto Manfredi, Enrico Ruffini, Davide Ottaviani, Libero Ciuffreda, Antonio Mussa, Riccardo Ferracini

**Affiliations:** 1 Center for Experimental Research and Medical Studies (CeRMS), University and AZO San Giovanni Battista, Turin, Italy; 2 Department of Human Anatomy and Histology, University of Bari, Bari, Italy; 3 Department of Medical Oncology, University and AZO San Giovanni Battista, Turin, Italy; 4 Department of Medical Oncology, Ospedale Civile, Asti, Italy; 5 Department of Toracic Surgery, AZO San Giovanni Battista, Turin, Italy; 6 Department of Orthopaedics, AZO San Giovanni Battista, Turin, Italy; New York University School of Medicine, United States of America

## Abstract

**Background:**

Interleukin-7 (IL-7) is a potent regulator of lymphocyte development, which has also significant effects on bone; in fact it is a potent osteoclastogenic factor. Some human solid tumors produce high IL-7 levels, suggesting a potential IL-7 role on tumor development and progression.

**Methodology:**

We studied 50 male patients affected by solid tumors, and their blood samples were collected at tumor diagnosis. PBMCs were isolated and cultured with/without IL-7 to study its influence on osteoclastogenesis. Serum and cell culture supernatant IL-7 levels were measured by ELISA. The quantitative analysis of IL-7 expression on T and B cells was performed by Real-Time PCR.

**Principal Findings:**

Serum IL-7 levels were highest in osteolytic cancer patients, followed by cancer patients without bone lesions, and then healthy controls. We showed the IL-7 production in PBMC cultures and particularly in monocyte and B cell co-cultures. A quantitative analysis of IL-7 expression in T and B cells confirmed that B cells had a high IL-7 expression. In all cell culture conditions, IL-7 significantly increased osteoclastogenesis and an anti-IL-7 antibody inhibited it. We demonstrated that IL-7 supports OC formation by inducing the TNF-α production and low RANKL levels, which synergize in promoting osteoclastogenesis.

**Conclusions:**

We demonstrated the presence of high serum IL-7 levels in patients with bone metastasis, suggesting the use of serum IL-7 level as a clinical marker of disease progression and of bone involvement. Moreover, we showed the capability of IL-7 to stimulate spontaneous osteoclastogenesis of bone metastatic patients and to induce osteoclastogenesis in cancer patients without bone involvement. These findings add further details to the disclosure of the mechanisms controlling bone metastasis in solid tumors.

## Introduction

Interleukin-7 (IL-7) is a pleiotropic immune regulatory protein predominantly produced by stromal cells and by cells at the inflammatory sites [1]. IL-7 is fundamental for the early development of lymphocytes and is a regulator of peripheral T cell homeostasis by modulating the expansion of peripheral T-cell populations in states of T cell depletion [2]. The production of IL-7 by some human solid tumors suggests its potential impact on the process of tumorigenesis [3], [4], but it is unclear how IL-7 is involved in solid tumor development and progression. IL-7 stimulates the progression of some types of lymphomas and leukaemias [5], [6] and in non-small cell lung cancer, it was reported that IL-7 targeted gene therapy may be effective in modifying host anti-tumor responses [7]. Recent studies on breast cancer describe a quantitative association between the IL-7 signalling complex and some clinico-pathological parameters: there is a trend towards a higher expression of IL-7 and molecules of its signalling pathway in breast cancer patients with poor prognosis [4]. Moreover, IL-7/IL-7R mRNA was detected in different tumors, such as colorectal [8], renal [9], lung and central nervous system cancers [10].

IL-7 is involved in the control of osteoclastogenesis, however depending on the model considered, it displays either an inhibitory or an activator effect on OCs [11], [12]. The OCs derive from cells of the monocytic-macrophagic lineage, which fuse to form bone-resorbing cells in the presence of Macrophage Colony Stimulating Factor (M-CSF) and Receptor Activator of Nuclear factor kB Ligand (RANKL) [13] or Tumor Necrosis Factor-α (TNF-α) [14], [15]. Weitzmann et al. demonstrated that systemic administration of IL-7 stimulates osteoclastogenesis through T cells by RANKL and TNF-α [16]. In addition, they showed that TNF-α produced by T cells synergises with RANKL increasing bone destruction in murine models of estrogen deficiency [17], [18].

The addition of exogenous cytokines is necessary to stimulate osteoclastogenesis in patients without osteolysis and in healthy controls. Previously, we demonstrated that peripheral blood mononuclear cells (PBMCs) from cancer patients with osteolysis differentiate spontaneously into OCs. In addition, we showed that in patients with osteolysis the activation of osteoclastic precursors depends on circulating tumor cells or on factors released from the tumor site, such as TNF-α [15].

To better identify the factors involved in the spontaneous osteoclastogenesis, present in bone metastatic patients, we focused our studies on IL-7, since it have been associated with haematological malignancies and inflammatory diseases characterized by a local and/or systemic bone loss [19]–[21].

IL-7 involvement in the bone metastasis formation and in the spontaneous osteoclastogenesis of cancer patients adds a further detail to the mechanisms of bone resorption and might be useful to design novel therapeutic approaches for treatment and prevention of bone metastasis.

## Results

### IL-7 levels dosed in supernatants and sera are higher in cancer patients than in healthy controls

**Figure 1:**
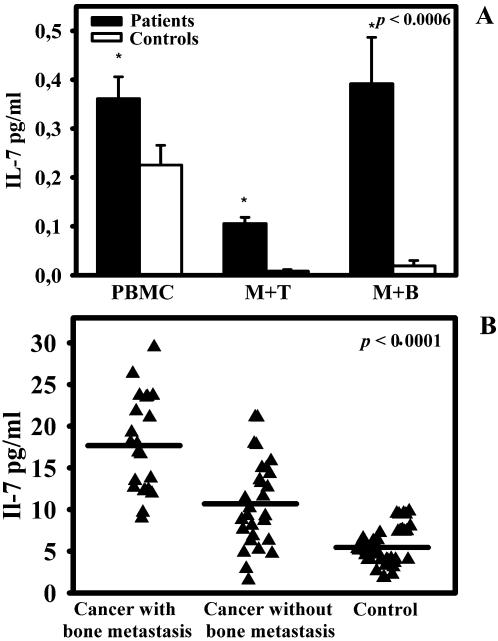
IL-7 levels in cell culture medium and serum. IL-7 levels in cancer patients with/without bone metastases and in healthy controls were analysed by ELISA. At day 5, supernatants of patients' PBMC cultures and monocyte plus T and B cell co-colture had IL-7 levels higher than healthy controls (A). Bone metastatic patients had significantly higher serum levels of IL-7 compared to patients without bone metastasis and healthy controls (B). Samples were assayed in duplicate and data were expressed as mean values.

We measured the IL-7 levels in 25 patient and healthy control PBMC supernatants, collected at days 5 and 10 of culture. We observed that IL-7 levels were significantly higher in patients than in healthy controls at day 5, *p*<0,0006 (Fig. 1A). At day 10, the IL-7 concentration was comparable to the one at day 5, both in patients and in healthy controls (data not shown). To identify the IL-7 source we performed co-colture experiments of monocytes plus B or T cells and we measured its levels in supernatants at day 5 and 10. We showed that IL-7 levels were significantly higher in cancer patients' co-coltures of both monocytes plus T or B cells than in healthy controls (*p*<0,0006) and remained nearly steady between day 5 and 10. In our model, the purified B cell population is responsible for IL-7 production: co-coltures of monocytes and B cells showed the highest IL-7 levels. Both in PBMC cultures and in monocytes plus T and B cells derived from patients without bone lesions we measured low levels of IL-7, comparable to healthy controls.

Besides, we tested serum levels of IL-7 in 50 patients affected by solid tumors with or without metastasis at the time of diagnosis. Fig. 1B showed that IL-7 serum levels were significantly higher in patients with bone metastasis compared to patients without bone lesions and healthy controls, *p*<0,0001. The mean IL-7 value was 17,68±1,24 pg/ml for osteolytic patients, 10,73±0,10 pg/ml for patients without bone lesion and 5,56±0,30 pg/ml for healthy controls. We examined the potential differences in serum IL-7 levels according to the various types of tumor and specifically we considered prostate cancer, NSCLC and SCLC. We identified significant differences in the mean value of IL-7 (*p*≤0,02), which were 19.89±6.30 pg/ml for prostate cancer, 14.6±5.95 pg/ml for NSCLC and 7.56±4.09 pg/ml for SCLC.

### Evaluation of IL-7 expression in T and B cells with (RQ-PCR)

**Figure 2:**
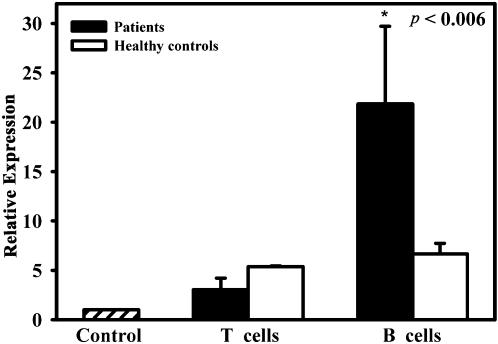
Quantitative analysis of IL-7 expression in T and B cells. IL-7 expression was evaluated by RQ-PCR using 2^−ΔΔCt^ method. B cells derived from cancer patients expressed significantly higher levels of IL-7 compared to T cells. Both T and B cells from healthy controls expressed less IL-7 than patients. The control bar represents the negative control: IL-7 expression in H522 cell line. Data are means±SE of nine independent experiments.

We analysed the expression of IL-7 on freshly purified T and B cells by RQ-PCR. The analyzed transcripts exhibited high linearity amplification plots (r>0.99) and the efficiencies of both PCR reactions were quite similar. As shown in Fig. 2, we did not measure significant differences in the expression of IL-7 in both patients and healthy controls' T cells, while in healthy controls' B cells the IL-7 expression was five-fold lower than in patients. The relative expression of IL-7 was seven-fold higher in B cells than in T cells in cancer patients. We did not appreciate any significant differences in IL-7 expression in cancer patients with or without bone metastasis (Fig. 2).

### Effects of IL-7 on osteoclastogenesis

**Figure 3:**
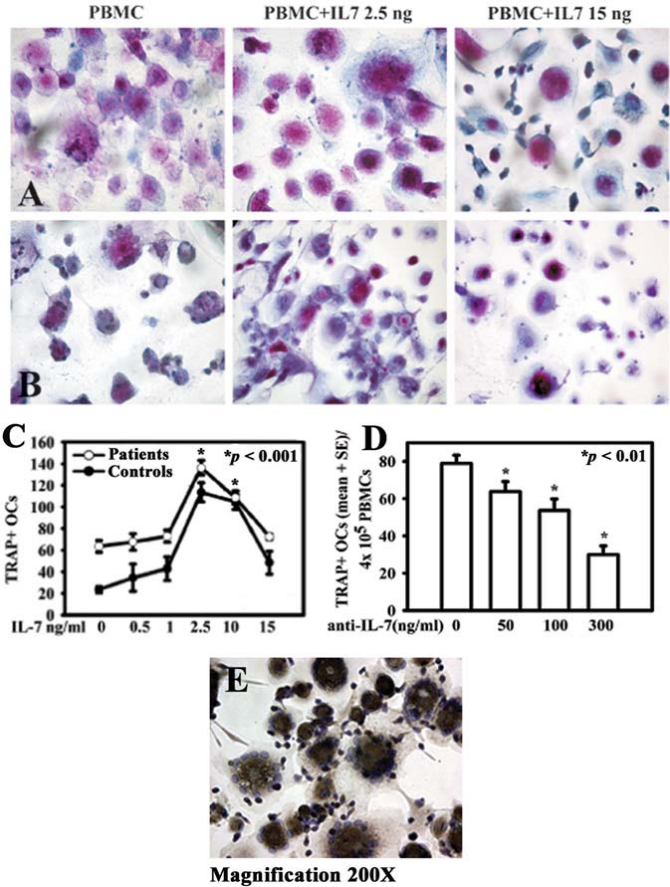
Effect of IL-7 on osteoclastogenesis *in vitro.* TRAP^+^ multinucleated cells were identified as OCs at the end of culture in both bone metastatic patients **(line A)** and healthy controls **(line B)**. PBMC cultures were plated and stimulated with IL-7 (0,5–1–2,5–10–15 ng/ml). The osteoclastogenesis significantly increased at 2,5 ng/ml of IL-7 compared to the unstimulated condition **(C)**. The anti-IL-7 antibody added in cell culture determined a dose-dependent inhibition of spontaneous osteoclastogenesis in bone metastatic patients **(D)**. Multinucleated/TRAP positive cells, derived both from culture with and without IL-7, expressed also α_V_β_3_ **(E)**.

The OC differentiation was verified by the presence of multinucleated/TRAP positive cells from cancer patient and healthy control PBMCs (Fig. 3A–B lines, respectively). PBMCs were cultured at different doses of IL-7, that resulted in significantly increased osteoclastogenesis with 2,5 and 10 ng/ml of IL-7 in osteolytic patients and in osteoclastogenesis stimulation for patients without bone lesions and healthy controls, *p*<0,0001. At 15 ng/ml, IL-7 had not a stimulatory effect on osteoclastogenesis (Fig. 3C). By adding a neutralizing anti-IL-7 antibody on osteolytic patients' PBMC, we observed a dose-dependent inhibition of spontaneous osteoclastogenesis, *p*<0,01 (Fig. 3D). We characterized OCs also for the expression of α_V_β_3_, a typical OC marker. Both OCs derived from culture with and without IL-7 expressed α_V_β_3_, as shown in Fig. 3E.

**Figure 4:**
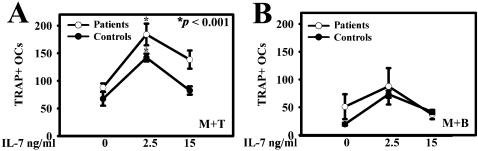
IL-7 stimulates osteoclastogenesis in co-coltures of monocytes and T or B cells. The different subpopulations were isolated from PBMCs and co-coltures of monocytes and T or B cells (M+T; M+B) were made. By adding 2,5 ng/ml of IL-7 in co-coltures we observed an increase in osteoclastogenesis, which was statistically significant for T cells **(A)** and not for B cells **(B)**. At 15 ng/ml of IL-7, there were not significant effects in both the co-coltures.

The ability of IL-7 to induce OC formation was tested in cultures of purified monocytes alone and in co-cultures with purified T or B cells treated or untreated with IL-7 at 2,5 and 15 ng/ml. Our results showed that monocytes alone were not able to support significant OC formation in either the presence or absence of IL-7, in both cancer patients and healthy controls (data not shown). Moreover, we demonstrated that co-cultures of monocytes and T cells both from bone metastatic patients and healthy controls resulted in a significant induction of OC formation by adding 2,5 ng/ml compared to basal condition, *p* < 0,001, while the dose of 15 ng/ml did not elicit a significant stimulatory effect on osteoclastogenesis (Fig. 4A). Co-cultures of monocytes and B cells in presence of IL-7 showed an increase in OC number, but it was not statistically significant (Fig. 4B). In all culture conditions, we observed a comparable osteoclastogenesis in patients without bone metastases and in healthy controls (data not shown).

Since we showed that spontaneous osteoclastogenesis depends on contact between monocytes and lymphocytes [15], we investigated whether T and B cells were necessary for IL-7-dependent OC differentiation. We cultured purified monocytes with IL-7-treated conditioned medium from T and B cells and we did not observe OC differentiation (data not shown). Thus, cell-to-cell interaction between lymphocytes and monocytes are required for solid tumor dependent osteoclastogenesis.

### IL-7 stimulates OC resorbing activity

**Figure 5:**
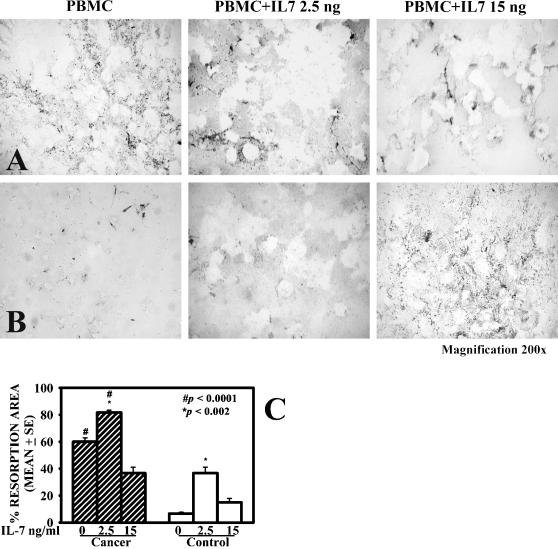
IL-7 increases OC bone resorbing activity. PBMC resorption activity with/without IL-7 were showed for bone metastatic patients **(line A)** and healthy controls **(line B)**. The basal resorbing activity of OCs derived from patients was significantly higher compared to healthy controls, ^#^ *p*≤0,0001 **(C)**. The percentage of resorption area was significantly increased with 2,5 ng/ml of IL-7, both in patients and in healthy controls, *p≤0,002 **(C)**. At 15 ng/ml IL-7 did not elicit a significant variation of resorption area. The data represent the means±SE from ten independent experiments.

Since we showed that IL-7 had effects in stimulating osteoclastogenesis, we verified whether there was a similar stimulatory action on bone resorbing activity. By analysing the resorption area on biocoated slices, we demonstrated that in presence of IL-7 at 2,5 ng/ml the OCs increased the bone resorbing activity of osteolytic patients and stimulated the healthy controls' resorption (Fig. 5A–B lines, respectively). The basal resorption activity of OCs derived from patients was significantly higher compared to healthy controls, ^#^ *p*≤0,0001 (Fig. 5C). After stimulation with IL-7 at 2,5 ng/ml, the bone resorbing activity of OCs was significantly augmented both in patients and in healthy controls, *p≤0,002 (Fig. 5C). At 15 ng/ml of IL-7, we did not observe any statistically significant variation on bone resorbing areas.

### IL-7 affects TNF-α and RANKL release in culture

**Figure 6:**
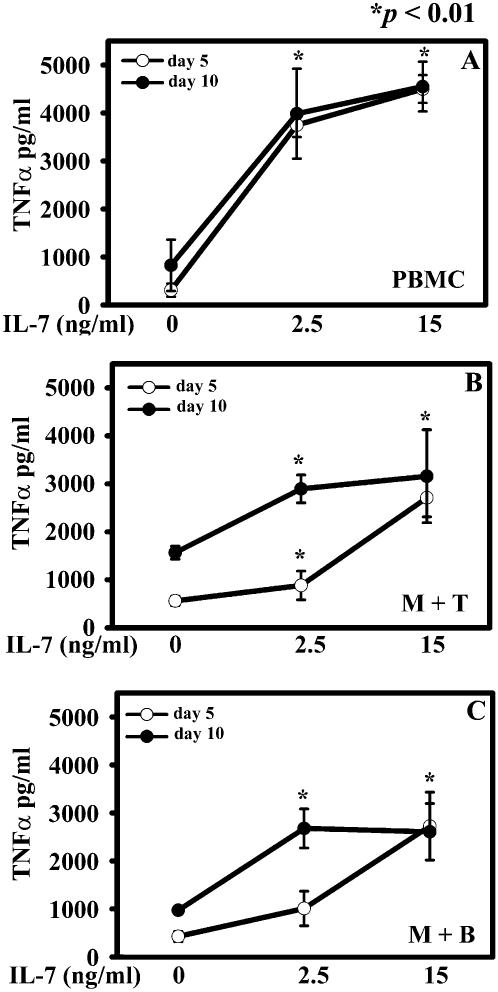
IL-7 up-regulates TNF-α production in cell culture media. PBMC and monocytes plus T or B cells were cultured in absence or presence of IL-7. Supernatants were collected at days 5 and 10 to dose TNF-α. PBMCs showed the same TNF-α basal level, which increased at 2,5 ng/ml of IL-7 and reached the maximum value at 15 ng/ml, **(A)**. Monocytes plus T cells (M+T) **(B)** and monocytes plus B cells (M+B) **(C)** had a TNF-α basal level higher at day 10 than at day 5, but both supernatants showed the peak value at 2,5 ng/ml of IL-7, and remained roughly costant at 15 ng/ml. The data were means±SE of 25 supernatants collected at day 5 and 10 for both patients and healthy controls.

We assessed the IL-7 ability to induce TNF-α and RANKL secretion in PBMC cultures and monocyte plus T or B cell co-coltures at day 5 and 10. The basal levels of TNF-α were similar in PBMC media at day 5 and 10 and they significantly increased by adding 2,5 ng/ml of IL-7, *p*<0,002 (Fig. 6A). Co-cultures of monocytes and T or B cells showed basal TNF-α levels higher at day 10 than at day 5. Monocytes plus T cells displayed an IL-7 dose-dependent increase of TNF-α level both on days 5 and 10, *p*<0,01 (Fig. 6B). At 15 ng/ml of IL-7, TNF-α values reached a plateau level both in PBMC cultures and in monoytes plus T cell co-coltures (Fig. 6A–B). At day 5, co-coltures of monocytes and B cells showed a significant enhancement of TNF-α amount at 15 ng/ml, whereas at day 10 we measured a significant increase in TNF-α levels at 2.5 ng/ml, that was unchanged at 15 ng/ml of IL-7, *p*≤0,01 (Fig. 6C).

**Figure 7:**
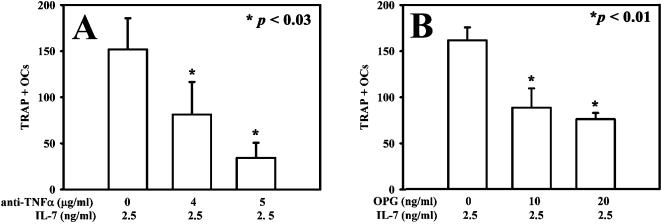
Effect of anti-TNF-α and OPG on IL-7-induced osteoclastogenesis. The addition of both OPG and anti-TNF-α caused a dose-dependent inhibition of IL-7-induced osteoclastogenesis. The anti-TNF-α antibody caused a strong osteoclastogenesis inhibition, about 90% **(A)** while OPG caused a 50% inhibition **(B)**.

We found low RANKL levels in culture media collected from PBMC and monocyte plus T or B co-coltures. In particular, in the IL-7 treated cells there were not significant differences compared to unstimulated controls (Table 1). To further study the involvement of TNF-α and RANKL we cultured cancer patients' PBMC in presence of IL-7 and a neutralizing anti-TNF-α antibody or osteoprotegerin (OPG) in different concentrations. Both the inhibitors determined a dose-dependent inhibition of bone resorbing activity (data not shown) and of osteoclastogenesis, that was stronger in cultures treated with anti-TNF-α (Fig. 7A) than with OPG (Fig. 7B). Thus, our data demonstrated that IL-7 supports osteoclastogenesis by inducing mainly the TNF-α production and low levels of RANKL.

**Table 1:**
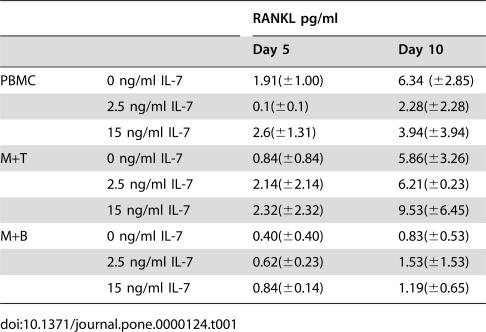
RANKL levels in cell culture media.

		RANKL pg/ml	
		Day 5	Day 10
PBMC	0 ng/ml IL-7	1.91(±1.00)	6.34 (±2.85)
	2.5 ng/ml IL-7	0.1(±0.1)	2.28(±2.28)
	15 ng/ml IL-7	2.6(±1.31)	3.94(±3.94)
M+T	0 ng/ml IL-7	0.84(±0.84)	5.86(±3.26)
	2.5 ng/ml IL-7	2.14(±2.14)	6.21(±0.23)
	15 ng/ml IL-7	2.32(±2.32)	9.53(±6.45)
M+B	0 ng/ml IL-7	0.40(±0.40)	0.83(±0.53)
	2.5 ng/ml IL-7	0.62(±0.23)	1.53(±1.53)
	15 ng/ml IL-7	0.84(±0.14)	1.19(±0.65)

## Discussion

Over the last years, many studies investigated the cross-talk between immune system and bone, and IL-7 appeared as an important factor in these interactions [12], [22], [23]. IL-7 is known to be a powerful inducer of T and B lymphopoiesis, moreover many studies link IL-7 to inflammatory diseases [21], [24], [25] and bone loss in estrogen deficiency conditions [22], [26]. Our study focused on IL-7 involvement in the spontaneous osteoclastogenesis occurring in cancer patients with osteolytic metastasis. We showed significantly higher IL-7 levels in supernatants derived from PBMC cultures and co-coltures of monocytes and T or B cells of cancer patients compared to healthy controls. Our data describe for the first time the presence of elevated IL-7 levels in sera of male patients affected by solid tumors, in fact the only data available in literature, concerning IL-7 serum levels in patients affected by solid tumors are related to gynecologic cancers [27]–[29], while elevated serum IL-7 levels were previously detected in other pathologies with bony involvement, such as multiple myeloma [19], rheumatoid arthritis [30] and systemic juvenile rheumatoid arthritis [31]. It is interesting to note that patients affected by bone metastatic cancer had an IL-7 value higher than non-bone metastatic ones, and these latter showed an intermediate IL-7 serum value between bone metastatic patients and healthy controls. The high IL-7 serum levels might be interpreted as a signal of disease progression towards the metastatic phenotype in some non-bone metastatic tumor patients. The analysis of the available data showed that NSCLC and prostate cancer had a statistically significant higher IL-7 serum levels compared to SCLC cases. However, in all the specific cancer groups (NSCLC, prostate cancer and SCLC) we found that patients with bone metastasis had higher IL-7 levels than patients without osteolysis. These findings might infer that in different tumor types the increase of IL-7 serum levels might be useful for early diagnosis of metastatic bone lesions. Literature data support the IL-7 production by different tumor types [4], [8], [10] apart from stromal cells in bone microenvironment. Previously reported data showing the role of B cells in IL-7 production [6], [19], [23], [32], were confirmed by our finding that B cells produced IL-7 and this production was higher than T cell one.

In this study we demonstrated that IL-7 at 2,5 ng/ml increased osteoclastogenesis in patients' cultures of PBMCs and co-coltures of monocytes plus T or B cells, while at the dose of 15 ng/ml, IL-7 did not affect osteoclastogenesis. To explain this result we hypotesize that, at high doses, IL-7 stimulates a differentiation switch from OCs towards dendritic cells since in cultures with 15 ng/ml of IL-7 we observed only few OCs and mainly undifferentiated macrophages or cells with dendritic cells morphology. This hypothesis is supported by some studies showing that IL-7 regulates dendritic cells differentiation from PBMC cultures [33], [34]. Since literature data link IL-7 to TNF-α and RANKL-dependent osteoclastogenesis [16], [19], [35], we measured their levels in culture media. In detail, we showed that IL-7 enhances in a dose-dependent manner TNF-α levels in PBMC culture media and in co-coltures of monocytes and T or B cells. We hypothesize that TNF-α levels reached maximal values in the PBMC cultures probably due to a cooperative release of TNF-α by monocytes [21], T and B cells stimulated by IL-7. We observed a little and not statistically significant dose-dependent increase of RANKL release from day 5 to day 10 of culture compared to unstimulated condition. According to literature data [36], [37] we think that these low levels of RANKL are synergistic with TNF-α in promoting osteoclastogenesis. In fact we observed that both anti-TNF-α antibody and OPG caused a dose-dependent inhibition of IL-7-induced osteoclastogenesis. The anti-TNF-α antibody reached an OC inhibition plateau at 90% while OPG treatment of cell cultures reached a plateau of OC differentiation inhibition at 50%. Further increase in OPG concentrations, in an attempt to reach an higher level of inhibition, was found to be toxic for the cultured cells. This observation suggests that in our model TNF-α might have a stronger role in OC formation than RANKL; it is conceivable that both factors may synergize in OC differentiation.

These last results demonstrate that TNF-α plays a fundamental role in IL-7-induced osteoclastogenesis in patients affected by solid tumor. Moreover, the pivotal role exerted by TNF-α in our systems was also supported by the fact that in freshly isolated T and B cells from patients we did not find RANKL expression, while TNF-α was strongly present. TNF-α enhancement by IL-7 is also present in other pathological conditions, characterized by a local and/or systemic bone loss, such as rheumatoid arthritis [30] or postmenopausal osteoporosis [38].

In conclusion, we propose a new physiopathologic mechanism with a key role of IL-7 in the formation of solid tumor bone metastasis. We demonstrated that IL-7, produced mainly by B cells in cell culture, directly sensitizes T cells to produce pro-osteoclastogenic factors, such as TNF-α and RANKL, and enhances spontaneous osteoclastogenesis. It could be useful to analyse a large number of patients for each different tumor type, in order to set an IL-7 cut off value, representative of a warning threshold for clinicians. This could allow to follow the bone metastatic disease both at diagnosis and during treatments.

## Material and Methods

### Patients

Samples from peripheral blood (PB) were obtained from 50 male patients affected by solid tumors and 50 healthy controls, matched for age and sex. The main clinical characteristics of patients, aged from 50 to 84 years (median 65±11,01 SD), are listed in Table 2. The patients' samples were collected at diagnosis to exclude biases, including recent chemo/hormono-therapy and pre-existing or non-related bone pathologies. We decided not to include breast cancer patients to avoid bias deriving from women treated with steroids or hormonal therapies.

**Table 2:**
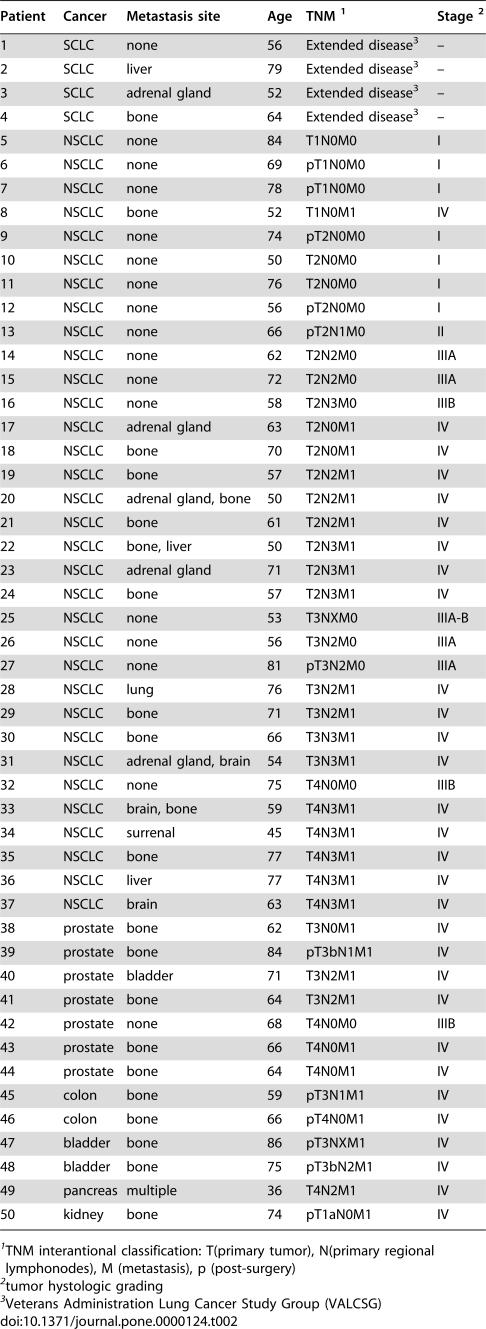
Patient characteristics.

Patient	Cancer	Metastasis site	Age	TNM 1	Stage 2
1	SCLC	none	56	Extended disease^3^	–
2	SCLC	liver	79	Extended disease^3^	–
3	SCLC	adrenal gland	52	Extended disease^3^	–
4	SCLC	bone	64	Extended disease^3^	–
5	NSCLC	none	84	T1N0M0	I
6	NSCLC	none	69	pT1N0M0	I
7	NSCLC	none	78	pT1N0M0	I
8	NSCLC	bone	52	T1N0M1	IV
9	NSCLC	none	74	pT2N0M0	I
10	NSCLC	none	50	T2N0M0	I
11	NSCLC	none	76	T2N0M0	I
12	NSCLC	none	56	pT2N0M0	I
13	NSCLC	none	66	pT2N1M0	II
14	NSCLC	none	62	T2N2M0	IIIA
15	NSCLC	none	72	T2N2M0	IIIA
16	NSCLC	none	58	T2N3M0	IIIB
17	NSCLC	adrenal gland	63	T2N0M1	IV
18	NSCLC	bone	70	T2N0M1	IV
19	NSCLC	bone	57	T2N2M1	IV
20	NSCLC	adrenal gland, bone	50	T2N2M1	IV
21	NSCLC	bone	61	T2N2M1	IV
22	NSCLC	bone, liver	50	T2N3M1	IV
23	NSCLC	adrenal gland	71	T2N3M1	IV
24	NSCLC	bone	57	T2N3M1	IV
25	NSCLC	none	53	T3NXM0	IIIA-B
26	NSCLC	none	56	T3N2M0	IIIA
27	NSCLC	none	81	pT3N2M0	IIIA
28	NSCLC	lung	76	T3N2M1	IV
29	NSCLC	bone	71	T3N2M1	IV
30	NSCLC	bone	66	T3N3M1	IV
31	NSCLC	adrenal gland, brain	54	T3N3M1	IV
32	NSCLC	none	75	T4N0M0	IIIB
33	NSCLC	brain, bone	59	T4N3M1	IV
34	NSCLC	surrenal	45	T4N3M1	IV
35	NSCLC	bone	77	T4N3M1	IV
36	NSCLC	liver	77	T4N3M1	IV
37	NSCLC	brain	63	T4N3M1	IV
38	prostate	bone	62	T3N0M1	IV
39	prostate	bone	84	pT3bN1M1	IV
40	prostate	bladder	71	T3N2M1	IV
41	prostate	bone	64	T3N2M1	IV
42	prostate	none	68	T4N0M0	IIIB
43	prostate	bone	66	T4N0M1	IV
44	prostate	bone	64	T4N0M1	IV
45	colon	bone	59	pT3N1M1	IV
46	colon	bone	66	pT4N0M1	IV
47	bladder	bone	86	pT3NXM1	IV
48	bladder	bone	75	pT3bN2M1	IV
49	pancreas	multiple	36	T4N2M1	IV
50	kidney	bone	74	pT1aN0M1	IV

1 TNM interantional classification: T(primary tumor), N(primary regional lymphonodes), M (metastasis), p (post-surgery)

2 tumor hystologic grading

3 Veterans Administration Lung Cancer Study Group (VALCSG)

Healthy controls showed a normal bone metabolism (evaluated by bone densitometry) and were not under medications, such as steroids that could increase osteoclastogenesis. Informed consent from patients and healthy controls was obtained to comply with institutional policies.

### Cell cultures

PBMCs, collected from cancer patients and healthy controls, were isolated after centrifugation over a density gradient, Lymphoprep (Nycomed Pharma, Norway) and cultured in α-MEM, supplemented with 10% FBS, penicillin 100 U/ml and streptomycin 100 µg/m (Cambrex, Walkersville MD). To obtain fully differentiated human OCs, PBMCs were cultured with/without rhIL-7 at different doses (0,5–1–2,5–10 and 15 ng/ml), R&D Systems (Abingdon, UK). Culture supernatants were collected on days 5 and 10, when medium was either refreshed and supplemented or not with IL-7. For some experiments, patient PBMCs were cultured with different doses of anti-IL7 (50–100–300 ng/ml), R&D Systems (Abingdon, UK). Cultures were stopped after 15 days, mature OCs were identified as multinucleated cells containing three or more nuclei and positive for the expression of TRAP (Tartrate-resistant acid phosphatase, Sigma Aldrich, St. Louis MO) and α_V_β_3_ (vitronectin receptor) kindly provided by Prof. G. Tarone. To evaluate bone resorbing activity, for 21 days PBMCs were cultured on Biocoat Osteologic bone cell culture system (BD Biosciences Bedford, MA) with/without IL-7, then cells were removed and resorption lacunae were identified by light microscopy.

For some experiments PBMCs were cultured in presence of increasing concentration of a neutralizing anti-TNFα (4–5 µg/ml) antibody and osteoprotegerin (OPG 10–20 ng/ml), PeproTech (London, UK).

### Isolation of monocytes, T and B cells

Monocytes, T and B cells were isolated from PBMCs by an immunomagnetic selection using MACs microbeads (Miltenyi Biotec GmbH, Bergisch Gladbach, Germany), according to the manufacturer's instructions. The purity of monocytes, T and B cells, obtained after separation, was 95–98%, as assessed by flow cytometry. Isolated cells were plated according to the human physiological concentrations: 10% monocytes and 30% lymphocytes (70% for T and 30% for B) with/without IL-7.

### ELISA (Enzyme-Linked Immunosorbent Assay)

The amount of IL-7 in cell culture supernatant and serum was determined by ELISA kit (sensitivity was 0,156 to 20 pmol/L and 0 to 16 pmol/L, respectively. R&D Systems, Abingdon UK). TNF-α (Biosource, Nivelles Belgium) and RANKL (Biomedica Gruppe, Wien Austria) ELISA kit were performed on culture supernatants, the sensitivity range was 0 to 1500 pmol/L and 0 to 8 pmol/L, respectively.

### Quantitative analysis of IL-7 gene expression

Total RNA was extracted from T and B cells and the first-strand cDNA synthesis was performed as previously described [15]. Quantitative analysis of IL-7 was performed with Real-Time Quantitative PCR (RQ-PCR) using β-actin as housekeeping control. RT-PCR was carried out using the iCycler iQ™ system (Bio Rad, CA, USA). PCR primers and TaqMan probes were designed using Primer Express v1.0 software and synthesized by Applied Biosystems (Warrington, UK). TaqMan probe specific for IL-7 (5′-TGAGAGTGTTCTAATGGTCAGCATCGATCAAT-3′) and for β-actin (5′- CGTCACCAACTGGGACGACATGG-3′) were both labelled at the 5′ end with 6-carboxy fluorescein (FAM) and the 3′ end with 6-carboxy-tetrametil rhodamine (TAMRA). The IL-7 primer sequences were: sense 5′-TGAAGGTAAAGATGGCAAACAA-3′; antisense 5′-CAATTTCTTTCATGCTGTCCAA-3′. The β-actin primer sequences were: sense 5′-CCCTGAAGTACCCCATCGA-3′; antisense 5′- AAGGTGTGGTGCCAGATTTTC-3′. Reactions for IL-7 and β-actin quantification were performed in a 25 µl final volume with 2 µl of sample cDNA, 1X iQ Supermix (Bio Rad), 0,4 µM of each primer and 0,4 µM of the IL-7 probe. The amplification conditions for quantization were: 95°C for 15 minutes and 50 cycles of 95°C for 15 seconds and 58°C for 1 minute.

We tested a series of cell lines for the IL-7 expression and we chose , as positive control, MG63, an osteosarcoma cell line that expressed at high level IL-7. As negative control we used H522, a non-small cell lung cancer cell line. Relative IL-7 quantization in T and B lymphocytes, expressed as –fold variation over control (H522), was calculated after determination of the difference between C_T_ (threshold cycle) of the given gene A (IL-7) and that of the calibrator gene B (β-actin) using the 2^−^ΔΔ^CT^ method [39]. C_T_ values are means of duplicate measurements. To validate the use of the 2^−ΔΔCT^ method, serial dilutions (ranging from 10^0^ to 10^−5^) of MG63 cDNA were used to obtained a standard curve.

### Statistical analyses

Statistical analyses were performed with the Statistical Package for the Social Sciences (spssx/pc) software (SPSS, Chicago, IL). We compared the results by means of student's paired *t* test. The results were considered statistically significant for *p*<0,05.
